# Antiplatelet Therapy Aims and Strategies in Asian Patients with Acute Coronary Syndrome or Stable Coronary Artery Disease

**DOI:** 10.3390/jcm11247440

**Published:** 2022-12-15

**Authors:** Chor-Cheung Tam, Hung-Fat Tse

**Affiliations:** 1 Division of Cardiology, Queen Mary Hospital, The University of Hong Kong, Hong Kong, China; 2 Cardiology Division, Department of Medicine, Li Ka Shing Faculty of Medicine, The University of Hong Kong, Hong Kong, China

**Keywords:** antiplatelet agent, bleeding, clopidogrel, dual antiplatelet therapy, individualized medicine, ischemia, P2Y_12_ receptor antagonist, prasugrel, risk assessment, ticagrelor

## Abstract

Dual antiplatelet therapy (DAPT) has been the mainstay treatment to reduce ischemic events, such as myocardial infarction or stroke, in patients with coronary artery disease (CAD). The development of potent P2Y_12_ inhibitors (ticagrelor and prasugrel) has helped to further reduce ischemic events, particularly among high-risk patients. Meanwhile, the evolution of newer generations of drug-eluting stents are also improving outcomes of percutaneous coronary intervention. Research studies on antiplatelet therapy in recent years have focused on balancing ischemic and bleeding risks through different strategies, which include P2Y_12_ inhibitor monotherapy, escalation and de-escalation, and extended DAPT. Because results from the large number of clinical studies may sometimes appear conflicting, this review aims to summarize recent advances, and demonstrate that they are aligned by a general principle, namely, strategies may be adopted based on treatment aims for specific patients at several time points. Another aim of this review is to outline the important considerations for using antiplatelet therapy in Asian patients, in whom there is a greater prevalence of *CYP2C19* loss-of-function mutations, and a common increased risk of bleeding, despite high platelet reactivity (the so-called “East Asian Paradox”).

## 1. Introduction: Ischemic and Bleeding Risks

Aspirin, an irreversible cyclooxygenase (COX)-1 inhibitor, is currently the most widely used medication worldwide [1]. For decades, aspirin has been given to patients with cardiovascular (CV) and cerebrovascular conditions to reduce ischemic events, such as myocardial infarction (MI) and stroke, by diminishing platelet activity. Dual antiplatelet therapy (DAPT) was introduced in the mid-1990s, wherein aspirin is given in combination with a purinergic P2Y_12_ receptor inhibitor (P2Y_12_i; e.g., ticlopidine) [2]. Together, they provide improved antithrombotic efficacy by blocking both the COX-1 and adenosine diphosphate-dependent pathways for platelet aggregation [3]. Studies have repeatedly shown that DAPT reduces both the risk of acute thrombotic events, as well as long-term ischemic recurrence from atherosclerotic plague progression [4].

Because antiplatelet therapy (APT) reduces platelet response to vascular damage, an increase in the potency, dosage, and/or duration of APT also inevitably increases the patient’s risk of bleeding. This has been observed in the results of large-scale studies involving tens of thousands of patients. In other words, APT cannot reduce both ischemic and bleeding risks; rather, it poses a technological limitation that has yet to be overcome by innovations. Therefore, the balance between ischemic and bleeding risks has become the core subject of investigation in many recent trials. When prescribing APT, such a balance must be carefully and individually determined and monitored.

**Figure 1:**
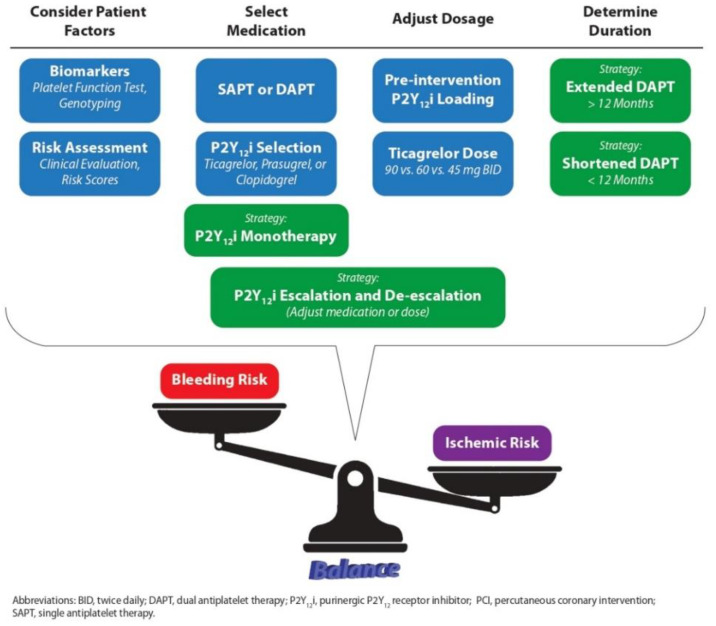
Schematic representation of various important considerations in antiplatelet therapy for patients with acute coronary syndrome or stable coronary artery disease, which have been the subjects of major clinical studies and literature discussions in recent years. Underlying these considerations is the critical notion of balancing ischemic risk and bleeding risk.

In planning for an APT, besides assessing ischemic and bleeding risks, there is a wide range of factors to consider. Figure 1 illustrates the major considerations that have undergone robust research in recent years.

## 2. APT Aims and Strategies

In the past decade, the introduction of newer potent P2Y_12_ inhibitors (e.g., ticagrelor and prasugrel) has helped to further reduce the occurrence of ischemic events in coronary artery disease (CAD) patients [5]. Meanwhile, the development of new generations of drug-eluting stents, such as biodegradable polymer stents, also appears to have lowered the thrombotic risks following a percutaneous coronary intervention (PCI), when compared with the older bare metal stents [6]. In view of the improved APT potency and stent safety, the balance between ischemic and bleeding risks must also be managed in further detail.

Physicians working in the area may often wonder, why do results from the large number of clinical studies appear to be conflicting? For example, while some trials (e.g., PEGASUS-TIMI 54 [7]) suggest better outcomes with an extended DAPT duration, others support shortened DAPT (e.g., DAPT-STEMI [8]). Other trials suggest switching from DAPT to P2Y_12_i monotherapy by dropping aspirin (e.g., TWILIGHT [9]), or to a different P2Y_12_i dose or agent (e.g., HOST-REDUCE POLYTECH-ACS [10] and TOPIC [11]). To answer this question, it is essential to realize that these trials target different patient populations and are concerned with different research questions and objectives.

Depending on the specific aims of APT, different strategies may be adopted (Table 1). Table 1 defines short-, medium-, and long-term APT as approximately <1 month, 1–12 months, and >12 months, which are arbitrary divisions that coincide with common designs of randomized controlled trials (RCTs) of APT. In practice, APT duration is often a moving target [12] that is contingent on patient factors and treatment tolerance. While, at hospital discharge, it may not be possible to determine a patient’s risk over time, risk assessment should be re-evaluated regularly [13].

In addition to the differences in medication strategy, the trials were conducted in different patient groups (e.g., those with acute coronary syndrome [ACS] or stable CAD [sCAD]) and regions (e.g., U.S., Europe, or the Asia-Pacific), using various measurement criteria (e.g., Thrombolysis in Myocardial Infarction [TIMI] or Bleeding Academic Research Consortium [BARC] bleeding criteria). This review aims to categorize the recent results, and layout an important conceptual framework that underlies these studies, namely, ischemic and bleeding risks may vary for different patients at different time points.

## 3. Short-to-Medium Term APT

### 3.1. Standard DAPT

Patients who recently had an ACS or are indicated for PCI have an elevated risk of experiencing an ischemic event (including recurrent MI), particularly in the first 30 days [14,15]. Although there are some suggestions of a decreasing trend in recurrent coronary hospitalization in recent years [16], the risk remains high, especially for patients with additional risk factors [17]. The aim of APT in these patients, by and large, is to aggressively reduce their ischemic risk, while avoiding any excessive increase in bleeding risk (Table 1).

Landmark RCTs that have established a standard DAPT duration of 12 months include CURE [18], PLATO [19], and TRITON [20], in which the ischemic benefits appeared to outweigh the bleeding risks (Table 2 and Table 3). For example, in PLATO [19], where ACS patients were randomized to receive ticagrelor 90 mg twice daily (BID) versus clopidogrel 75 mg once daily (QD), the occurrences of vascular death, MI, or stroke at 12 months were 9.8% versus 11.7%, respectively (*p* < 0.001), and the rates of major bleeding were 11.6% versus 11.2% (non-significant [N.S.]). All-cause deaths occurred in 4.5% versus 5.9% (*p* < 0.001) of patients in the two arms, respectively.

Recent Asian studies of 1-year DAPT in ACS patients, such as PHILO [21], TICAKOREA [22], and PRASFIT-Practice-II [23,24], reported somewhat lower rates of ischemic events. TICAKOREA [22] also reported significantly reduced bleeding rates for patients treated with clopidogrel versus ticagrelor. While these results might reflect the more recent and Asian clinical scenarios, these studies also had smaller sample sizes compared with the trials above. APT for Asian patients will be further discussed in Section 5.

### 3.2. P2Y_12_i Monotherapy

Hypothetically, P2Y_12_i monotherapy may provide two potential benefits over traditional DAPT: first, it may reduce bleeding while providing similar ischemic protection in the medium term; second, it reduces the medication burden in the longer term (e.g., when administered beyond 1 year).

Notable trials include TWILIGHT [9,26], SMART-CHOICE [28], and STOPDAPT-2 [29,30]. TWILIGHT demonstrated significantly reduced bleeding at 15 months in patients treated with ticagrelor monotherapy after 3 months of DAPT, compared with those who continued DAPT, both in the overall population (4.0% [ticagrelor alone] vs. 7.1% [ticagrelor + aspirin], *p* < 0.001) [26] and the ACS subgroup (3.6% [ticagrelor alone] vs. 7.6% [ticagrelor + aspirin], *p* < 0.001), but not in the sCAD subgroup (4.8% [ticagrelor alone] vs. 6.2% [ticagrelor + aspirin]; N.S.) [9].

In the Asian studies SMART-CHOICE [28] and STOP-DAPT2 [29], with PCI patients, switching to P2Y_12_i monotherapy also reduced bleeding without compromising ischemic event prevention. However, STOP-DAPT2-ACS [30], where ACS patients were switched from DAPT to clopidogrel monotherapy, did not achieve noninferiority, and there was a marginal increase in the major composite ischemic endpoint (2.8% vs. 1.9%, hazard ratio [HR] = 1.50, 95% confidence interval [CI]: 0.99–2.26), including a HR of 1.91 (95% CI: 1.06–3.44) for MI. One explanation could be that 1 month of DAPT was too short for ACS patients, whose conditions are more severe and unstable, and clopidogrel resistance might also have affected ischemic outcomes.

### 3.3. DAPT Escalation and De-Escalation, including Shortened DAPT

Another strategy is de-escalation, where DAPT continues at a reduced dose or duration, or with a less potent P2Y_12_i. Both “unguided” (by randomized allocation only) and “guided” (e.g., by platelet function test [PFT] or genotyping) de-escalation approaches have produced favorable results. A recent network meta-analysis [46] compared APT trials that shortened DAPT with those that reduced P2Y_12_i dosage or potency (total 29 trials; 50,602 patients), and found no difference in all-cause death between the two. Reducing P2Y_12_i dosage or potency was favored in terms of trial-defined net adverse CV events (NACE; risk ratio [RR] = 0.87, 95% CI: 0.70–0.94), but not with respect to bleeding (RR = 1.54, 95% CI: 1.07–2.21). However, because some of the sample sizes in the escalation and de-escalation studies were relatively small, and most were open-label, adjudicator-blinded studies, there could potentially be some effects of patient selection, as well as bias in the reporting of both physician- and patient-reported clinical outcomes. More large-scale studies are required for further comparison.

It is worth noting that the time of de-escalation chosen in these trials vary in aggressiveness, from 1, 3 to 6 months after starting DAPT, i.e., when ischemic and bleeding risks remain high to becoming more stable. While these trials generally demonstrated a reduction in bleeding events without increasing ischemic events significantly, in real-life, the time chosen for de-escalation will depend on the patient’s characteristics and evolving risks.

#### Guided Escalation and De-Escalation

Currently, two kinds of test are available for helping to select patients for the different APT strategies: PFT and genotyping. PFT measures platelet activation levels and may be performed at baseline and during APT [47]. Different laboratory techniques may be used, including light transmission, electrical impedance, and flow cytometry [47]. The *RPFA-*VerifyNow^®^ *P2Y12* test is a point-of-care whole blood test for monitoring clopidogrel resistance; results are expressed as P2Y_12_ reaction units (PRU) [47]. Genotyping identifies cytochrome P450 loss-of-function (LOF) mutations, which are associated with clopidogrel resistance because they reduce the liver’s ability to metabolize clopidogrel into its active form [48].

In ANTARTIC [38], depending on PFT results, patients receiving DAPT could be escalated from prasugrel 5 mg QD to 10 mg QD (for those with high platelet reactivity [HPR]) or de-escalated to clopidogrel 75 mg QD (for those with low platelet reactivity). However, the trial failed to achieve superiority over DAPT with prasugrel 5 mg QD. In TROPICAL-ACS [39] and POPular Genetics [40], noninferiority was demonstrated for guided de-escalation from a potent P2Y_12_i to clopidogrel based on PFT results. PATH-PCI [42] escalated patients with high platelet maximum aggregation rate (>55%) from clopidogrel to ticagrelor, and produced a significant net clinical benefit.

In a meta-analysis [49] of guided-DAPT, encompassing 11 RCTs (six PFT-guided and five genotype-guided trials) and three observational studies (all genotype-guided studies) with 20,743 patients, guided APT was associated with reduced trial-defined major adverse CV events (MACEs; RR = 0.78, *p* = 0.015), CV death (RR = 0.77, *p* = 0.049), MI (RR = 0.76, *p* = 0.021), stent thrombosis (RR = 0.64, *p* = 0.011), stroke (RR = 0.66, *p* = 0.010), and minor bleeding (RR = 0.78, *p* = 0.003), but not all-cause death and major bleeding. The authors noted that, generally, guided escalation was associated with a reduction in ischemic risks without safety tradeoffs, whereas guided de-escalation was associated with bleeding reductions without efficacy tradeoffs [49].

TAILOR-PCI [41] enrolled 5,302 patients to receive genotype-guided or conventional DAPT. *CYP2C19* carriers in the genotype-guided arm received ticagrelor, and all other patients received clopidogrel. In a primary analysis of 1,849 *CYP2C19* LOF carriers, composite CV death, MI, stroke, stent thrombosis, and severe recurrent ischemia occurred in 4.0% (35/903) and 5.9% (54/946) of patients in the genotype-guided and conventional arms, respectively, but the difference did not reach statistical significance (*p* = 0.06). None of the 11 prespecified secondary endpoints, including major or minor bleeding, demonstrated statistical significance, except marginally for stent thrombosis (*p* = 0.05).

Nevertheless, an updated meta-analysis [50] of 11 RCTs (11,740 patients) on genotype-guided APT vs. standard treatment demonstrated significant reductions across all reported efficacy outcomes, including trial-reported MACEs (RR = 0.60, *p* = 0.001), all-cause death (RR = 0.70, p = 0.02), CV death (RR = 0.71, *p* = 0.02), MI (RR = 0.53, *p* < 0.0001), stroke (RR = 0.64, *p* = 0.04), stent thrombosis (RR = 0.63, *p* = 0.01), and target vessel revascularization (RR = 0.79, *p* = 0.003). Differences in all bleeding outcomes were non-significant: BARC types 2,3,5: RR = 0.87, *p* = 0.13; BARC types 3,5: RR = 1.14, *p* = 0.44; TIMI major: RR = 1.05, *p* = 0.81; TIMI minor: RR = 1.04, *p* = 0.88. Of note, the subgroup analysis suggested that genotype-guided APT was more likely to reduce MACEs in populations that consist of more ACS or Chinese patients [50].

Because point-of-care PFT is common, and genotyping results can be produced within a few days (in POPular Genetics, the median time between blood collection and genotyping result was 4 h only [51]), guided escalation and de-escalation may be performed quite readily, even within the first 2 weeks after PCI, as in the trials. However, Angiolillo et al. [4] cautioned that patients who are de-escalated to clopidogrel could in fact have HPR, and because 7–14 days of maintenance clopidogrel is required after de-escalation to assess platelet function, they can be subject to an increased risk of thrombosis.

## 4. Long-Term APT

### 4.1. Long-Term DAPT

Studies on MI recurrence generally suggest that, in 30-day survivors of acute MI, mortality rates plateau at about 3 years after the first index MI [52]. To prevent long-term ischemic events, several large-scale studies have investigated the efficacy and safety of extending DAPT from 1 year to about 3 years, most notably the DAPT [43] and PEGASUS TIMI-54 [7] trials. The DAPT trial [43] reported a 1.6% absolute reduction in all-cause death, MI, or stroke after 30 versus 12 months of DAPT with prasugrel or clopidogrel, which was coupled with a 0.9% absolute increase in moderate or severe bleeding according to the GUSTO (Global Use of Streptokinase and Tissue plasminogen activator to Open occluded coronary arteries) criteria.

PEGASUS [7] recruited patients who had a prior MI 1–3 years previously. Extended DAPT with ticagrelor plus aspirin achieved a 1.1% (ticagrelor 60 mg BID vs. aspirin alone, *p* = 0.004) or 1.2% (ticagrelor 90 mg BID vs. aspirin alone, *p* = 0.008) absolute reduction in CV death, MI, or stroke at 36 months, which was accompanied by a 1.2% or 1.5% absolute increase in TIMI major bleeding, for the two ticagrelor doses respectively (both *p* < 0.001). A post-hoc subgroup analysis of PEGASUS [53] illustrated that in patients with no bleeding risk indicators and ≥2 ischemic risk indicators (59% of 13,938 patients), ticagrelor significantly reduced the primary composite efficacy endpoint of CV death, MI, or stroke by 1.9% (*p* = 0.0024), and TIMI major bleeding (primary safety endpoint) only by 1.0% (*p* < 0.001). Given a moderate increase in bleeding, extended DAPT would likely benefit those who have elevated ischemic risks (e.g., impaired renal function, large atherosclerotic burden, multiple stents) and relatively low bleeding risks (e.g., young age; See Section 5).

THEMSIS-PCI [44] recruited patients with sCAD and diabetes mellitus, and found that, among those who underwent PCI, 3.3 years of ticagrelor (mostly at the lower 60-mg BID dose) led to a 1.3% absolute decrease in CV death, MI, or stroke, and a 0.9% increase in TIMI major bleeding. The significant ischemic benefit was not observed in patients without PCI.

### 4.2. Long-Term P2Y_12_i Monotherapy

Trials have also considered long-term P2Y_12_i monotherapy. GLOBAL LEADERS [31] demonstrated no significant differences between 1-month DAPT plus 23-month ticagrelor monotherapy versus 24-month DAPT, both in terms of ischemic and bleeding events, but these results were not sufficient for establishing superiority. The pre-specified subgroup analysis [54] revealed that BARC type 3 or 5 bleeding occurred in 1.95% versus 2.68% of ACS patients (*p* = 0.037), compared with 2.13% versus 1.62% in sCAD patients (*p* = 0.081), while differences in the primary endpoint of all-cause death or new Q-wave MI remained non-significant. In the ACS subgroup, there was a significant reduction in all-cause death, new Q-wave MI, and BARC type 3 or 5 bleeding when taken together (rate ratio = 0.81, *p* = 0.029). Although the superiority hypothesis was not sustained overall, the subgroup analysis suggests that ACS patients may still benefit from ticagrelor monotherapy following abbreviated DAPT. In the post-hoc landmark analysis of GLOBAL-LEADERS [55], which included patients who were event-free at 12 months, the second year of ticagrelor monotherapy demonstrated lower composite all-cause death, MI, or stroke compared with aspirin monotherapy (1.9% vs. 2.6%, log-rank *p* = 0.014, adjusted *p* = 0.022) that was driven by reduced MI (0.7% vs. 1.2%, *p* = 0.003). The authors also noted that the difference in BARC type 3 or 5 bleeding (0.5% vs. 0.3%, log-rank *p* = 0.051, adjusted *p* = 0.005) was significant only after adjustment for characteristics of patients excluded from the second-year analysis due to clinical events or nonadherence.

HOST-EXAM [33] enrolled PCI patients who were event-free after 6–18 months of prior DAPT. After another 24 months, compared with aspirin monotherapy, patients who received clopidogrel monotherapy had a reduced composite outcome of all-cause death, non-fatal MI, stroke, ACS re-admission, and BARC type ≥ 3 bleeding (5.7% vs. 7.7%, *p* = 0.003). One caution is that while both ischemic and bleeding endpoints decreased, all-cause deaths remained comparable (1.9% vs. 1.3%, *p* = 0.101).

### 4.3. Long-Term Anticoagulant plus Aspirin

COMPASS [45] investigated whether low-dose rivaroxaban, alone or in combination with aspirin, would be more effective for secondary CV prevention than aspirin alone. The trial recruited 27,395 patients with sCAD and/or peripheral arterial disease, of whom 62% had previous MI and 21% had heart failure. Patients who were already using anticoagulants were excluded, including those with atrial fibrillation (AF) receiving rivaroxaban at the standard dosage.

Participants were randomized to rivaroxaban plus aspirin, rivaroxaban alone, or aspirin alone. The trial was stopped at a mean follow-up of 23 months for superiority of the rivaroxaban plus aspirin combination. Compared with aspirin alone, there was a 1.3% absolute reduction in CV death, MI, or stroke, together with a 1.2% increase in modified ISTH (International Society on Thrombosis and Haemostasis) bleeding, which included hospitalized bleeding. Detailed analysis [56] also showed a significant reduction in stroke occurrences in the rivaroxaban plus aspirin group over the aspirin alone group (0.9% vs. 1.6% per year, *p* < 0.0001). There were significantly fewer cardioembolic strokes (*p* = 0.006) and embolic strokes of undetermined source (*p* = 0.006) in the rivaroxaban plus aspirin arm, compared with aspirin alone (secondary analysis) [57]. Niessner et al. [58] commented that subclinical AF might have underlain such results, as AF can be quite prevalent among peripheral arterial disease patients. During the 23-month follow-up, 49 patients (0.2% of 27,395) were diagnosed with AF [57].

## 5. Personalized Assessment

### 5.1. HBR Patients

Traditionally, to control for confounders and heterogeneity, APT trial recruitment often excludes patients with unstable bodily conditions that are not directly related to their CAD, including any risk of major bleeding, prior stroke, and the need for long-term oral anticoagulant use. As researchers realize the core importance of balancing between ischemic and bleeding risks in APT, more studies are addressing patients who fall into the “high bleeding risk” (HBR) category. Tools such as the PRECISE-DAPT score [59] (>25 points) and Academic Research Consortium for High Bleeding Risk (ARC-HBR) criteria [60] (one major or two minor criteria) have also been developed for identifying HBR patients.

Two recent international studies investigated DAPT duration for HBR patients. MASTER-DAPT [36] was a large-scale RCT powered to detect noninferiority in NACEs and MACEs, and superiority in major or clinically relevant bleeding. The XIENCE Short DAPT program [37] comprised three prospective, multicenter, non-randomized single-arm cohorts, which were compared using propensity score stratification. Criteria for HBR in these two studies varied, and included major bleeding history, stroke history, hematological disorders, and old age. In these two studies, 1 month of DAPT produced similar ischemic outcomes but reduced bleeding events, when compared with 3 months of DAPT. A MASTER-DAPT sub-analysis [61] also showed that BARC type 2, 3, or 5 bleeding was reduced in the 1-month DAPT arm, regardless of PCI complexity.

Some major APT trials have also conducted subgroup analysis on HBR patients. In the pre-specified TWILIGHT-HBR analysis [62], 17.2% of patients (1064 of 6178) met the ARC-HBR criteria. Compared with ticagrelor plus aspirin, ticagrelor monotherapy reduced BARC type 2, 3, or 5 bleeding in both the HBR (6.3% vs. 11.4%, *p* = 0.004) and non-HBR (3.5% vs. 5.9%, *p* < 0.001) subgroups. For BARC type 3 or 5 bleeding (i.e., more severe bleeding), there was a larger absolute risk reduction in the HBR group than the non-HBR group (−3.5% vs. −0.5%, *p* = 0.008). The key secondary endpoint of death, MI or stroke was similar between the two treatment arms, regardless of HBR status. In a post-hoc HBR subgroup analysis of STOP-DAPT2 [63], 1,054 of 3,009 patients (35%) were at HBR, according to the ARC-HBR criteria. The results showed consistent effects across the HBR and non-HBR subgroups, of 1-month DAPT followed by 11-month clopidogrel monotherapy versus 12-month DAPT. In line with TWILIGHT-HBR, there was also a numerically greater reduction in major bleeding in the STOP-DAPT2 HBR subgroup, compared with the non-HBR subgroup. In Chinese populations, 4-year post-hoc analysis of HBR (440 of 2737; 16%) patients from the I-LOVE-IT 2 trial [64] showed comparable efficacy and safety between 6- and 12-month DAPT. However, compared with non-HBR patients, HBR patients were associated with elevated risks of BARC type 3 or 5 (3.0% vs. 1.5%, *p* = 0.03), stroke (9.1% vs. 3.8%, *p* < 0.001), all-cause death (5.7% vs. 3.1%, *p* = 0.008), and NACE (31.8% vs. 26.0%, *p* = 0.01).

A meta-analysis [65] of six RCTs and three propensity-matched studies (i.e., the three XIENCE sub-studies [37]) compared ≤3-month DAPT with 6–12-month DAPT in 16,848 trial-defined HBR patients. The risks of ischemic events were similar, including MI (odds ratio [OR] = 1.16, 95% CI: 0.94–1.44), whereas major bleeding was lower with ≤3-month DAPT (OR = 0.68, 95% CI: 0.51–0.89). However, the authors noted a numerically higher incidence of late stent thrombosis (>30 days to 1 year) in their subgroup analysis, and suggested that, although newer stents are associated with lower late stent thrombosis rates, further investigations will be needed.

To minimize the decrease in ischemic protection for HBR CAD patients, besides optimizing the shortened DAPT duration, other studies have investigated the use of different stent types. (Conversely, when deciding on the appropriate APT for HBR patients, stent type may also be taken into consideration.) LEADERS FREE [66] and ONYX ONE [67] used similar sets of 13 criteria for determining HBR, including age ≥ 75 years (64% in LEADERS FREE; 62% in ONYX ONE), planned long-term oral anticoagulant use (36% in LEADERS FREE; 39% in ONYX ONE), and/or renal impairment (creatinine clearance < 40 mL/min; 19% in LEADERS FREE; 15% in ONYX ONE). In both studies, patients received only 1 month of DAPT, followed by aspirin alone or P2Y_12_i alone thereafter. In LEADERS FREE [68], at 2 years, with a population that included 42% ACS patients [66], the primary safety composite endpoint of cardiac death, MI, or stent thrombosis occurred in 12.6% of patients fitted with polymer-free drug-coated stents, versus 15.3% of those fitted with bare metal stents (*p* = 0.039). Clinically driven target-lesion revascularization was performed in 6.8% and 12.0% of the two arms, respectively (*p* < 0.0001). BARC types 3–5 bleeding occurred in 8.9% and 9.2% of patients (N.S.). In ONYX ONE, in which 52% were ACS patients [69], at 2 years [67], the primary safety composite endpoint of cardiac death, MI, or stent thrombosis occurred in 21.2% of those who received polymer-based stents, and in 20.7% who received polymer-free stents (N.S.). Target lesion failure (secondary effectiveness endpoint) happened in 22.1% versus 21.0% (N.S.), and BARC types 3–5 bleeding developed in 7.1% and 5.5% (N.S.) of the two groups of patients, respectively.

The 2018 European Society of Cardiology (ESC)/European Association for Cardio-Thoracic Surgery Guidelines on Myocardial Revascularization [70] offers a Class IIa, Level of Evidence (LoE) B recommendation for stented ACS HBR patients (with PRECISE-DAPT score ≥ 25) to discontinue P2Y_12_i after 6 months. For sCAD HBR patients, the recommended DAPT duration is 3 months (Class IIa, LoE A). The 2021 American College of Cardiology/American Heart Association/Society for Cardiovascular Angiography & Interventions [71] also offers a Class 2a, LoE A recommendation for shortened DAPT (1–3 months) in selected patients to reduce the risk of bleeding, with subsequent transition to P2Y_12_i monotherapy. In both guidelines, drug-eluting stents are generally strongly preferred over bare metal stents (Class I, LoE A), but there is not yet any specific recommendation on stent types in HBR patients.

### 5.2. Asian Patient Characteristics

Jeong [72] was among the first authors to formulate the notion of the “East Asian Paradox”: compared with Western patients, East Asian patients not only have higher risks of bleeding from APT, but also higher therapeutic levels of platelet reactivity. The difference in platelet reactivity may also influence ischemic risks, and some authors have observed that Asian studies tend to report low ischemic event rates [73]. Jeong derived the East Asian Paradox from two literature observations. First, whereas East Asian patients are more prone to warfarin-related intracranial hemorrhage compared with Western patients, an analogous pattern may be true for APT [72]. Second, in a platelet reactivity study, Japanese volunteers showed longer thrombotic occlusion time when compared with Western volunteers [74]. The East Asian Paradox suggests that the optimal APT regimens for East Asians may be different from those for Westerners and should be evaluated in further studies [72].

For example, in a meta-analysis of eight RCTs involving 37,775 ACS patients [75], DAPT de-escalation was associated with a significantly lower risk of major bleeding among East Asians (RR = 0.61, *p* = 0.048), but not among non-East Asians (RR = 0.73, *p* = 0.17). In both groups, the composite rates of all-cause death, MI, stroke, stent thrombosis, and revascularization were similar between the de-escalation and standard DAPT arms. An Asian expert consensus [76] suggested that demographics, comorbidities, and disease patterns in East Asian populations can influence therapeutic response and outcomes, which may help to explain this paradox.

**Figure 2:**
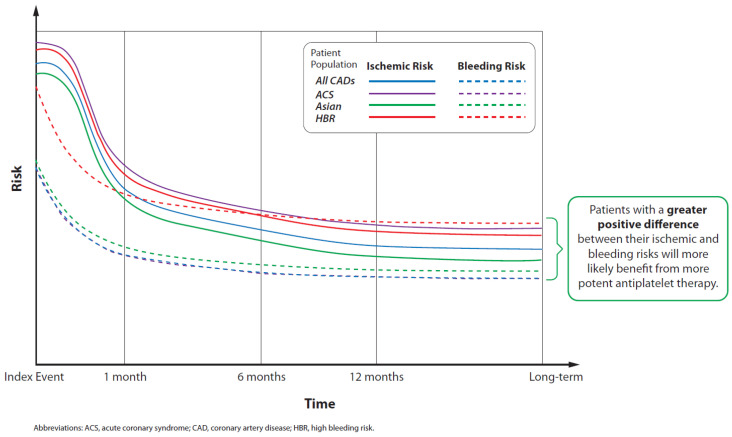
Schematic representation of usual changes in ischemic and bleeding risks over time (from an index time point) for several different populations, relative to all CAD patients (reference group). Note that the patient populations may overlap.

Figure 2 presents a theoretical representation of the general trends in ischemic and bleeding risks for different types of CAD patients, with reference to recent observations from the literature [12,73,76]. Immediately following the index event (e.g., PCI), all CAD patients tend to have both very high ischemic risk and high bleeding risk. These risks tend to gradually decline in the next 30 days, when the patient recovers from the ischemic event and/or surgery, and they continue to decline in the months that follow. The difference in magnitude between a patient’s ischemic and bleeding risks provides a therapeutic window for receiving APT to prevent ischemic events. Figure 2 also illustrates that ACS patients have higher ischemic risks than sCAD patients; patients in the HBR category have elevated bleeding risks compared with non-HBR patients; and Asian patients may be more prone to bleeding than Western patients.

### 5.3. Risk Assessment in Asian Patients

Various ischemic and bleeding risk assessment instruments have been validated in Asian populations. The DAPT score successfully stratified ischemic and bleeding risks in a pooled cohort of 12,223 Japanese patients [77]; however, the authors noted that ischemic event rates were low, even in patients with high DAPT scores. To evaluate bleeding risks, the PRECISE-DAPT score provides a standardized tool to predict out-of-hospital bleeding and has been validated in both Chinese and Korean populations [59,78]. Developed from the records of 32,057 patients from Hong Kong, the CARDIAC score [79] helps to predict the risk of major bleeding within 1 year after PCI, based on anticoagulation therapy, age, renal insufficiency, drop in hemoglobin levels, and baseline anemia. The reported discriminating power was an area-under-the-curve of 0.76, with an optimal cutoff that provides 63% sensitivity and 75% specificity. Physicians should also consider relevant clinical manifestations such as hemoglobin and creatinine levels, bruising and rectal bleeding. Table 4 provides a general list of common ischemic and bleeding risk factors, based on the ESC 2020 non-ST elevation ACS guidelines [80], the ARC-HBR consensus [60], and the DAPT [81], PRECISE-DAPT [82], and CARDIAC scores [79].

Because about 50% of East Asian patients have *CYP2C19* LOF mutations [83,84], which interferes with cytochrome P450 activation of clopidogrel, genotyping may be considered to test for mutation. A sequencing study [85] of 1,116 unrelated Hong Kong Chinese enrolled from 2012 to 2019 identified 29 actionable pharmacogenetic variants. At the gene level, *CYP2C19* was among several genes with the highest frequency of actionable phenotypes (57.2%), including 45.3% intermediate metabolizers and 12.0% poor metabolizers. Moreover, it should be noted that *CYP2C19* mutations only account for a fraction of the pharmacodynamic response to clopidogrel. In The ABCD-GENE risk score [86] for predicting HPR during clopidogrel treatment includes four clinical factors: age >75 years, body mass index >30 kg/m^2^, glomerular filtration rate <60 mL/min, and diabetes mellitus. Together with *CYP2C19* LOF alleles, these five factors produce a risk score with a C-statistic of 0.66 for all-cause death, stroke, or MI at 1 year [86].

Besides genotyping, point-of-care platelet reactivity test may also be performed to assess drug response while on APT. An international expert consensus [87] noted that PFT results and genetic markers have been reported to predict both thrombotic and bleeding events. Based on recent data, the panel agrees that, for patients on P2Y_12_i treatment, PFT results may provide useful prognostic data for CV risk prediction (both ischemic and bleeding events) after PCI. For ACS patients, although PFT is not recommended on a routine basis, for the purposes of treatment escalation or de-escalation, it may be considered in specific clinical scenarios. For sCAD patients, PFT is again not routinely recommended, but can be considered, in specific clinical scenarios, for switching to potent antiplatelet drugs in patients with increased thrombotic risk, and for determining which drug to keep upon DAPT cessation.

Table 5 provides a brief summary of key patient considerations for whether to reduce APT duration.

### 5.4. Common P2Y_12_i Drug Interactions

Some authors noted that HPR may sometimes be attributable to potential drug–drug interactions. For example, rifampicin induces CYP2C19 activity, whereas ketoconazole inhibits CYP3A4, leading to increased and decreased clopidogrel activation, respectively [88]. Conversely, clopidogrel may have perpetrator potentials, such as on cerivastatin and repaglinide by inhibiting CYP2C8 activity [89,90]. Presumably, drug–drug interactions may have more clinically significant effects on patients who have high or low platelet reactivity levels than those with normal levels, as had been suggested in trial patients who received atorvastatin and DAPT [91]. Of note, meta-analyses demonstrated that the co-administration of morphine and potent P2Y_12_i increased both platelet reactivity [92] and residual platelet reactivity [93]. This may be particularly relevant to the acute setting.

Observational studies have suggested some interaction effects between proton pump inhibitors and DAPT, with a high degree of heterogeneity [94]. While the only large-scale RCT on the prophylactic use of proton pump inhibitors in patients receiving clopidogrel demonstrated reduced upper gastrointestinal bleeding without increasing ischemic risks [95,96], guidelines vary in terms of patient selection for such prophylactic use [97].

In Asian patients, the use of traditional medicine (such as traditional Chinese medicine) has been shown in both animal and clinical studies to increase or decrease clopidogrel metabolism, by various proposed mechanisms [98]. Small exploratory trials on the concomitant use of traditional Chinese medicine and APT have been conducted to examine different hypotheses that include enhanced antiplatelet activity and reduced adverse effects [99].

### 5.5. Other Practical Considerations

There are a few caveats for interpreting the above trial results. First, trial designs often involve rather abrupt regimen changes in medication, dose adjustment, or discontinuation that might not be suitable for every patient. In practice, physicians may be able to implement changes more flexibly, coupled with close monitoring of risk factors and tolerance over time. In regions where patients have not been adequately represented in clinical trials, real-world studies may provide limited ideas on current practice patterns and outcomes. In Hong Kong, a retrospective matched cohort study of 6220 ACS patients treated in 14 hospitals between 2010 and 2017 [83] showed that potent P2Y_12_i use was associated with lower rates of ischemic stroke (HR = 0.57, *p* = 0.008) and thrombotic events (HR = 0.77, *p* = 0.001) compared with clopidogrel, while maintaining similar risks of intracranial hemorrhage (N.S.) and ISTH major bleeding (N.S.).

In Taiwan, a National Health Insurance Research Database study [100] of 27,339 acute MI patients (matched 1:8 ticagrelor: clopidogrel) reported lower all-cause death, acute MI, or stroke in the ticagrelor group versus the clopidogrel group (10.6% vs. 16.2%, HR = 0.78; 95% CI: 0.68–0.89), with similar major intracerebral or gastrointestinal bleeding (3.2% vs. 4.1%, HR = 0.73; 95% CI: 0.52–1.03 [N.S]).

## 6. Future Directions

### 6.1. Low-Dose Ticagrelor Monotherapy

Although studies on low-dose ticagrelor are still relatively uncommon, a meta-analysis [101] examined 16 trials including 1,629 ACS patients who received DAPT, of which 756 received low-dose ticagrelor: 484 received 90 mg QD, 240 received 45 mg BID, and 32 received 60 mg BID. Compared with clopidogrel 75 mg QD, low-dose ticagrelor significantly reduced CV death, MI, or stroke (OR = 0.39, 95% CI = 0.26–0.58, *p* < 0.01), without significantly increasing Study of Platelet Inhibition and Patient Outcomes (PLATO) major bleeding (OR = 1.16, *p* = 0.77). Also, in a patient-level meta-analysis [102] of six RCTs (24,096 patients) of P2Y_12_i monotherapy versus DAPT, P2Y_12_i monotherapy and DAPT showed similar composite risks of all-cause death, MI, or stroke. The risk of BARC type 3 or 5 bleeding was lower with P2Y_12_i monotherapy, when compared with DAPT (0.9% vs. 1.8%, *p* < 0.001), and particularly with newer P2Y_12_i (mainly ticagrelor).

When assessing patients’ platelet reactivity, Korean studies have adopted a range of 85–275 PRU, compared with the usual 85–208 (or sometimes 85–240) PRU range used in international studies [103]. This suggests a different therapeutic window for APT in Koreans compared with Western populations. Two small retrospective analyses [103,104] of on-treatment platelet reactivity assessed by the VerifyNow P2Y_12_ assay suggest that acute MI patients treated with standard-dose ticagrelor 90 mg BID resulted in average PRU values falling below 85. An upcoming phase 4 de-escalation trial will investigate the optimal dose (45 or 60 mg) of ticagrelor in Korean patients with acute MI (NCT05210595).

Ticagrelor monotherapy at a reduced dose of 60 mg BID (or even 45 mg BID) presents an attractive option for Asian patients, because of its potent, reversible antiplatelet activity, with the potential for less bleeding compared with the 90 mg BID dose. A recent 12-week prospective, single-center RCT [105] reported significantly improved brachial flow-mediated dilation in ACS patients treated with ticagrelor 60 mg BD monotherapy versus aspirin 100 mg OD alone: +3.48% vs. −1.26%, *p* < 0.001. Multi-omics signatures, including changes in amino acid and phospholipid metabolism and biosynthesis, were associated with the improved brachial artery flow-mediated dilation [105]. Future studies on low-dose ticagrelor, including monotherapy, are warranted.

### 6.2. Ticagrelor Reversal

To restore platelet activity in patients receiving ticagrelor, cardiac surgeons may give prophylactic platelet transfusion, fresh frozen plasma, and protamine infusion [106]. The use of an intravenous monoclonal antibody, bentracimab, for ticagrelor reversal was recently tested in a single-arm, prospective study with patients who required urgent surgery (n = 142) or had major bleeding (n = 8) [107]. The antiplatelet effects were reversed rapidly (within 5 to 10 min) and sustained for >24 h, with adjudicated hemostasis achieved in >90% of patients. This reversal agent, if available, may be particularly useful for patients with ST-elevation MI who require large surgical incisions and/or a prolonged operation period.

### 6.3. Comparing across APT Strategies

As emphasized early on in this review, it is a current technological limitation that APT cannot reduce both ischemic and bleeding risks. Hence, an APT strategy should be chosen depending on the specific treatment aim.

Nevertheless, sometimes more than one strategy appears feasible, and no direct comparative evidence is available. Indeed, while a plethora of trials have been conducted on the different APT strategies, head-to-head trials are lacking. Large-scale studies comparing APT strategies would be challenging to conduct, but highly informative. A recent meta-analysis of 30 extended, standard, and de-escalation APT RCTs supported the safety of two strategies: 3-month DAPT followed by ticagrelor monotherapy, as well as a short period of high potency DAPT followed by clopidogrel + aspirin [108]. Another meta-analysis of seven de-escalation trials favored early de-escalation of DAPT after 1 to 3 months to P2Y1_2_i monotherapy [109]. A network meta-analysis of 29 studies including 50,602 patients [46] (see also Section 3.3) calculated based on posterior probability the outcomes of various de-escalation strategies. Short DAPT followed by aspirin monotherapy generally led to increased trial-defined NACE; for example, when compared with short DAPT followed by P2Y_12_i monotherapy (RR = 1.22, 95% CI: 1.00–1.48). When compared with standard DAPT, short DAPT followed by P2Y_12_i monotherapy reduced NACE (RR = 0.85, 95% CI: 0.73–0.98), as did DAPT de-escalation to clopidogrel (RR = 0.77, 95% CI: 0.68–0.88) and DAPT de-escalation to halved dose (RR = 0.71, 95% CI: 0.54–0.93). These results should be interpreted with some caution because of the multiple comparisons, overall statistical complexity, and clinical heterogeneity.

Continued understanding and exploration of the molecular mechanisms of platelet aggregation may one day help to create antiplatelet agents that reduce both ischemic and bleeding risks. Meanwhile, the development of biomarkers (e.g., metabolomics) [110] and machine learning algorithms [111] may help to better predict ischemic risks, bleeding risks, and antiplatelet response in individual patients.

## 7. Conclusions

In recent years, the efficacy and safety of a spectrum of APT strategies, in addition to standard 1-year DAPT, have been investigated in numerous RCTs. These strategies include P2Y_12_i monotherapy, guided and unguided de-escalation, as well as extended DAPT. Because an optimal APT regimen hinges on a delicate balance between ischemic and bleeding risks, the selection of APT should be based on specific treatment aims, with consideration for evolving patient risk factors and time of treatment. Compared with Western populations, Asian patients may be more prone to *CYP2C19* LOF mutations, increased platelet reactivity, and bleeding. Bleeding risk scores, genotyping, PFT, and low-dose ticagrelor therapy are among some of the potentially useful tools available for Asian populations.

## Figures and Tables

**Table 1:** Treatment aims and strategies of antiplatelet therapy at various periods.

Patient Population	Risk	Treatment Aims at Different Phases		
		Short Term (<1 Month)	Medium Term (1–12 Months)	Long Term (>12 Months)
ACS	Ischemic	↓↓↓	Avoid unacceptable ↑	↓
	Bleeding	Avoid excessive ↑	↓	Avoid excessive ↑
sCAD	Ischemic	↓	Avoid unacceptable ↑	↓
	Bleeding	Avoid excessive ↑	↓	Avoid excessive ↑
Example Strategies		DAPT using a potent P2Y12i (ticagrelor or prasugrel) + aspirin	DAPT duration adjustment P2Y_12_i monotherapy De-escalation (P2Y12i dosage or potency)	Extended DAPT P2Y_12_i monotherapy Anticoagulant

Abbreviations: “↓”, decrease; “↑”, increase; ACS, acute coronary syndrome; DAPT, dual antiplatelet therapy; P2Y_12_i, purinergic receptor P2Y_12_ inhibitor; sCAD, stable coronary artery disease.

**Table 2:** Study designs of major international and Asian randomized controlled trials investigating the efficacy and safety of popular antiplatelet therapy strategies.

CAD Population	Prior Procedure	Region(s)	Experiment Strategy	Blinding	Trial Name	Comparison Arms	Study Duration (Months)	Sample Size	
								Experiment	Control
**Standard 1-year DAPT**									
***Landmark Trials***									
ACS (STEMI excluded)	Exclude PCI in past 3 months	International	1-year DAPT	Double-blind	CURE [18,25]	ASA (75–325 mg) ± clopidogrel (300 mg loading + 75 mg QD)	12	6259	6303
ACS	PCI	International (91.7% Caucasians)	1-year DAPT	Double-blind	PLATO [19]	Ticagrelor (180 mg loading + 90 mg BID) vs. clopidogrel (300–600 mg loading + 75 mg QD)	12	9333	9291
ACS	PCI: 99% CABG: 1%	International (92–93% Caucasian)	1-year DAPT	Double-blind	TRITON [20]	Prasugrel (60 mg loading + 10 mg QD) vs. clopidogrel (300 mg loading + 75 mg QD)	15	6813	6795
***Asian Trials***									
ACS	PCI: 85%	Japan, Taiwan, Korea	1-year DAPT	Double-blind	PHILO [21]	Ticagrelor (180 mg loading + 90 mg BID) vs. clopidogrel (300 mg loading + 75 mg QD)	12	401	400
ACS	Invasive management	Korea	1-year DAPT	Open-label, adjudicator-blinded	TICAKOREA [22]	Ticagrelor (180 mg loading + 90 mg BID) vs. clopidogrel (600 mg loading + 75 mg QD)	12	400	400
57% ACS	PCI	Japan	2-year DAPT	Not specified	PRASFIT-Practice II [23,24]	Observational only: Prasugrel (20 mg loading + 3.75 mg QD)	24	4155	
**P2Y_12_i Monotherapy**									
***Landmark Trial***									
65% ACS	PCI	International (23.0% enrolled from Asia)	3-month DAPT + 12-month P2Y_12_i monotherapy	Double-blind	TWILIGHT [9,26]	Ticagrelor (90 mg BID) ± ASA	15	NSTE-ACS:	
								2273	2341
								sCAD:	
								1281	1222
***Asian Trials***									
ACS	Bioresorbable polymer sirolimus-eluting stent	Korea	3-month DAPT + 9-month P2Y_12_i monotherapy	Unblinded	TICO [27]	3-month DAPT (ticagrelor 90 mg BID + ASA) + 9-month ticagrelor 90 mg BID, vs. 12-month DAPT with ticagrelor 90 mg BID	12	1527	1529
58% ACS	PCI (certain stent types)	Korea	3-month DAPT + 9-month P2Y_12_i monotherapy	Open-label, adjudicator-blinded	SMART-CHOICE [28]	3-month DAPT with P2Y_12_i (clopidogrel 75 mg QD, prasugrel 10 mg QD or ticagrelor 90 mg BID + ASA) + 9-month P2Y_12_i, vs. 12-month DAPT	12	1495	1498
38% ACS	PCI with cobalt chromium everolimus-eluting stent	Japan	1-month DAPT + 11-month P2Y_12_i monotherapy	Open-label, adjudicator-blinded	STOPDAPT-2 [29]	ASA + clopidogrel (75 mg QD) or prasugrel (3.75 mg QD) for 1 month, followed by clopidogrel (75 mg QD) alone, vs. ASA + clopidogrel (75 mg QD) for 12 months	12	1500	1509
ACS only				Open-label	STOPDAPT-2 ACS [30]			2078	2091
**Long-term P2Y_12_i Monotherapy**									
***Landmark Trial***									
47% ACS	PCI with DES	International	1-month DAPT + 23-month P2Y_12_i monotherapy	Open-label, adjudicator-blinded	GLOBAL LEADERS [31]	ASA + ticagrelor 90 mg BID for 1 month, followed by ticagrelor 90 mg BID alone for 23 months, vs. ASA + clopidogrel 75 mg QD (in sCAD patients) or ticagrelor 90 mg BID (in ACS patients) for 12 months, followed by ASA alone for 12 months	24	7980	7988
					GLASSY (GLOBAL LEADERS sub-study of top 20 enrolling sites) [32]			3794	3791
***Asian Trial***									
72% ACS; had 6–18 months prior DAPT	PCI	Korea	24-month P2Y_12_i monotherapy	Open-label, adjudicator-blinded	HOST-EXAM [33]	24-month clopidogrel (75 mg QD) alone vs. ASA (100 mg QD) alone	24	2710	2728
**Unguided De-escalation**									
***European Trials***									
ACS	PCI	France	1-month DAPT with ticagrelor or prasugrel + 11-month DAPT with clopidogrel	Open-label, adjudicator-blinded	TOPIC [11]	1-month ticagrelor (180 mg loading + 90 BID) or prasugrel (60 mg loading + 10 mg QD), followed by 11-month clopidogrel (75 mg), vs. 12-month ticagrelor or prasugrel	12	322	323
STEMI	PCI with second generation zotarolimus-eluting stent	Europe	6-month DAPT (at baseline) + 6-month ASA monotherapy	Open-label, adjudicator-blinded	DAPT-STEMI [8]	ASA ± P2Y_12_i (prasugrel: 60 mg loading + 10 mg QD; ticagrelor: 180 mg loading + 90 mg BID; or clopidogrel: 600 mg loading + 75 mg QD) for 6 months	18	432	438
***Asian Trials***									
ACS	PCI with durable or absorbable polymer DES	Korea	1-month DAPT + 11-month DAPT at reduced prasugrel dose	Open-label, adjudicator-blinded	HOST-REDUCE POLYTECH-ACS [10]	ASA + prasugrel (10 mg QD) for 1 month, followed by ASA + prasugrel (5 mg vs. 10 mg) for 11 months	12	1170	1168
STEMI and NSTEMI	PCI with DES	Korea	1-month DAPT with ticagrelor + 11-month DAPT with clopidogrel	Open-label, adjudicator-blinded	TALOS-AMI [34]	1-month ticagrelor (180 mg loading + 90 mg BID) + 11-month clopidogrel (75 mg QD), vs. 12-month ticagrelor (90 mg BID)	12	1349	1348
82% ACS	PCI with biodegradable polymer sirolimus-eluting stent	China	6-month DAPT	Assessor-blinded	I-LOVE-IT 2 [35]	6-month v. 12-month DAPT with clopidogrel (300 mg loading + 75 mg QD)	18	909	920
***HBR Patients***									
HBR; 48% ACS	PCI with biodegradable polymer sirolimus-eluting stent	International	1-month DAPT	Open-label, adjudicator-blinded	MASTER-DAPT [36]	1-month vs. ≥3-month DAPT (median: 193 days)	335 days	2295	2284
HBR; 35% ACS	PCI with cobalt chromium everolimus-eluting stent	International	1- or 3-month DAPT	Open-label, adjudicator-blinded	XIENCE Short DAPT [37]	1-month vs. 3-month DAPT (3 single-arm studies)	12	1392	1972
**Guided Escalation and De-escalation**									
***European Trials***									
ACS	PCI with stent	France	PFT-guided escalation or de-escalation	Open-label, adjudicator-blinded	ANTARTIC [38]	DAPT with prasugrel (5 or 10 mg QD) or clopidogrel 75 mg QD (monitoring group), vs. prasugrel 5 mg QD (conventional group)	12	442	435
ACS	PCI	Europe	PFT-guided de-escalation	Open-label, adjudicator-blinded	TROPICAL-ACS [39]	DAPT with 1-week prasugrel (5 or 10 mg QD) + 1-week clopidogrel (75 mg QD) + PFT-guided prasugrel or clopidogrel, vs. prasugrel (5 or 10 mg)	12	1304	1306
STEMI	PCI with stent	The Netherlands	Genotype-guided APT	Open-label, adjudicator-blinded	POPular Genetics [40]	DAPT with ticagrelor or prasugrel (*CYP2C19* carriers) or clopidogrel (noncarriers), vs. ticagrelor or prasugrel (standard group)	12	1242	1246
***Asian Trials***									
82% ACS	PCI	International (23% East Asians)	Genotype-guided APT	Open-label, adjudicator-blinded	TAILOR-PCI [41]	Aspirin 81 mg + ticagrelor (*CYP2C19* LOF carriers) or clopidogrel (noncarriers)	12	903 *	946 *
sCAD	PCI with DES	China	PFT-guided DAPT	Open-label, adjudicator-blinded	PATH-PCI [42]	DAPT with ticagrelor 90 mg BID (if maximum aggregation rate [MAR] > 55%) or 75 mg clopidogrel QD (if MAR ≤55%), vs. DAPT with clopidogrel 75 mg QD (standard group)	6	1123	1114
**Extended DAPT**									
***Landmark Trials***									
43% ACS	DES implantation	International (91.2% Caucasian)	30-month DAPT	Open-label, adjudicator-blinded	DAPT [43]	30-month vs. 12-month clopidogrel (75 mg QD) or prasugrel (5 or 10 mg QD)	30	5020	4941
Prior MI (1–3 years ago)	83% had PCI	International (86.6% Caucasian)	3-year DAPT	Double-blind	PEGASUS-TIMI 54 [7]	Ticagrelor 90 mg BID vs. ticagrelor 60 mg BID vs. placebo	36	7050 (90 mg)/7045 (60 mg)	7067
sCAD and DM	58% had PCI	International (22.9% Asians)	Long-term DAPT	Double-blind	THEMIS-PCI [44]	Ticagrelor (90 mg until May 2015, then 60 mg) + ASA vs. ASA alone	Median follow-up: 3.3 years	PCI:	
								5558	5596
								No PCI:	
								4061	4005
**Long-term Anticoagulant + ASA**									
***Landmark Trial***									
Stable atherosclerotic vascular disease (62% had previous MI)	--	International (12.6% Asians)	Rivaroxaban + ASA	Double-blind	COMPASS [45]	Rivaroxaban 2.5 mg BID + ASA, vs. rivaroxaban 5 mg BID alone, vs. ASA alone	Mean follow-up: 23 months (stopped for superiority of rivaroxaban + ASA combination)	9152 (2.5 mg)/9117 (5 mg)	9126

* Primary analysis (all *CYP2C19* loss-of-function carriers). Abbreviations: ACS, acute coronary syndrome; ASA, aspirin; BID, twice daily; CABG, coronary artery bypass graft; DES, drug-eluting stent; DM, diabetes mellitus; HBR, high bleeding risk; MI, myocardial infarction; NSTE, non-ST elevation; P2Y_12_i, purinergic P2Y_12_ receptor inhibitor; PCI, percutaneous coronary intervention; PFT, platelet function test; QD, once daily; sCAD, stable coronary artery disease; STEMI, ST-elevation myocardial infarction.

**Table 3:** Outcomes of major international and Asian randomized controlled trials investigating the efficacy and safety of popular antiplatelet therapy strategies.

Trial Name	Main Composite Ischemic Endpoint *	Main Ischemic Outcome (Experiment vs. Control) *	Main Bleeding Criteria *	Main Bleeding Outcome (Experiment vs. Control) *
**Standard 1-year DAPT**				
CURE [18,25]	CV death, MI, stroke	ASA ≤ 100 mg: 8.6% vs. 10.5%, RR = 0.81 (0.68–0.97) ASA 101–199 mg: 9.5% vs. 9.8%, RR = 0.97 (N.S.) ASA ≥ 200 mg: 9.8% vs. 13.6%, RR = 0.71 (0.59–0.85)	Significantly disabling, intraocular bleeding leading to significant loss of vision, or bleeding requiring transfusion of 2 or 3 units of red blood cells (or equivalent whole blood).	3.7% vs. 2.7%, RR = 1.4 (1.1–1.7)
PLATO [19]	Vascular death, MI, stroke	9.8% vs. 11.7%, *p* < 0.001	Fatal bleeding, intracranial bleeding, intrapericardial bleeding with cardiac tamponade, hypovolemic shock or severe hypotension due to bleeding and requiring pressors or surgery, a decline in the hemoglobin level ≥ 5.0 g/dL, or requiring transfusion of ≥ 4 units of red cells.	11.6% vs. 11.2% (N.S.)
TRITON [20]	CV death, MI, stroke	9.9% vs. 12.1%, *p* < 0.001	TIMI major	2.4% vs. 1.8%, *p* = 0.03
PHILO [21]	Vascular death, MI, stroke	9.0% vs. 6.3% (N.S.)	PLATO major	10.3% vs. 6.8% (N.S.)
TICAKOREA [22]	CV death, MI, stroke	9.2% vs. 5.8% (N.S.)	PLATO major + minor bleeding (clinically significant bleeding)	11.7% vs. 5.3%, *p* = 0.002
PRASFIT-Practice II [23,24]	CV death, MI, stroke, stent thrombosis	1-year: 1.6% 2-year: 3.3%	TIMI major	1-year: 1.0% 2-year: 2.7%
**P2Y_12_i Monotherapy**				
TWILIGHT [9,26]	All-cause death, MI, stroke	NSTE-ACS: 4.3% vs. 4.4% (N.S.) sCAD: 3.1% vs. 3.2% (N.S.)	BARC 2,3,5	NSTE-ACS: 3.6% vs. 7.6%, *p* < 0.001 sCAD: 4.8% vs. 6.2% (N.S.)
TICO [27]	Death, MI, stroke, stent thrombosis, target-vessel revascularization	2.3% vs. 3.4% (N.S.)	TIMI major	1.7% vs. 3.0%, *p* = 0.02
SMART-CHOICE [28]	All-cause death, MI, stroke	2.9% vs. 2.5%, *p* = 0.007 for noninferiority	BARC ≥ 2	2.0% vs. 3.4%, *p* = 0.02
STOPDAPT-2 [29]	CV death, MI, stroke, stent thrombosis	2.0% vs. 2.5%, *p* = 0.005 for noninferiority	TIMI major and minor	0.4% vs. 1.5%, *p* = 0.004 for superiority
STOPDAPT-2 ACS [30]		2.8% vs. 1.9%, HR = 1.50 (0.99–2.26)		0.5% vs. 1.2%, HR = 0.46 (0.23–0.94)
**Long-term P2Y_12_i Monotherapy**				
GLOBAL LEADERS [31]	All-cause death, MI	3.8% vs. 4.4% (N.S.)	BARC 3,5	2.0% vs. 2.1% (N.S.)
GLASSY (GLOBAL LEADERS sub-study of top 20 enrolling sites) [32]	All-cause death, MI, stroke, urgent revascularization	7.1% vs. 8.5%, *p* < 0.001 for noninferiority	BARC 3,5	2.5% vs. 2.5% (N.S.)
HOST-EXAM [33]	All-cause death, MI, stroke, re-admission to due ACS	3.7% vs. 5.5%, *p* = 0.003	BARC ≥ 3	1.2% vs. 2.0%, *p* = 0.035
**Unguided De-escalation**				
TOPIC [11]	CV death, stroke, urgent revascularization	9.3% vs. 11.5% (N.S.)	BARC ≥ 2	4% vs. 14.9%, *p* < 0.01
DAPT-STEMI [8]	Net clinical benefit composite (all-cause death, MI, stroke, revascularization, TIMI major bleeding): 4.8% vs. 6.6%, *p* = 0.004 for noninferiority			
HOST-REDUCE POLYTECH-ACS [10]	CV death, MI, ischemic stroke, stent thrombosis	1.4% vs. 1.8% (N.S.)	BARC ≥ 2	2.9% vs. 5.9%, *p* < 0.0007
TALOS-AMI [34]	CV death, MI, stroke	2.1% vs. 3.1% (N.S.)	BARC 2,3,5	3.0% vs. 5.6%, *p* = 0.001
I-LOVE-IT 2 [35]	Net adverse clinical and cerebral events composite (all-cause death, MI, stroke, BARC ≥ 3 bleeding): 12-month: 7.2 vs. 6.4% (N.S.) 18-month: 7.8% vs. 7.3% (N.S.)			
MASTER-DAPT [36]	All-cause death, MI, stroke	6.0% vs. 6.1% (N.S.)	BARC 2,3,5	6.5% vs. 9.4%, *p* < 0.001 for suepriority
XIENCE Short DAPT [37]	All-cause death or MI	1–12 months: 7.3% vs. 7.5% (N.S)	BARC ≥ 2	1–12 months: 7.6% vs. 10.0%, *p* = 0.012
**Guided Escalation and De-escalation**				
ANTARTIC [38]	CV death, MI, stroke, stent thrombosis, urgent revascularization	9% vs. 10% (N.S.)	BARC 2,3,5	20% vs. 21% (N.S.)
TROPICAL-ACS [39]	CV death, MI, stroke	3% vs. 3%, *p* = 0.01 for noninferiority	BARC ≥ 2	5% vs. 6% (N.S.)
POPular Genetics [40]	all-cause death, MI, stroke, stent thrombosis	2.7% vs. 3.3% (N.S.)	PLATO	9.8% vs. 12.5%, *p* = 0.04
TAILOR-PCI [41]	CV death, MI, stroke, stent thrombosis, severe recurrent ischemia	4.0% vs. 5.9%, *p* = 0.06 (N.S.)	TIMI major and minor	1.9% vs. 1.6% (N.S.)
PATH-PCI [42]	Net clinical adverse events composite (cardiac death, MI, stroke, stent thrombosis, urgent revascularization, BARC 2,3,5 bleeding): 5.1% vs. 7.5%, *p* = 0.023			
**Extended DAPT**				
DAPT [43]	All-cause death, MI, stroke	4.3% vs. 5.9%, *p* < 0.001	GUSTO moderate or severe	2.5% vs. 1.6%, *p* = 0.001
PEGASUS-TIMI 54 [7]	CV death, MI, stroke	7.85% vs. 7.77% vs. 9.04% Ticagrelor 90 mg vs. placebo: *p* = 0.008 Ticagrelor 60 mg vs. placebo: *p* = 0.004	TIMI major	2.60% vs. 2.30% vs. 1.06%, *p* < 0.001 for each dose vs. placebo
THEMIS-PCI [44]	CV death, MI, stroke	PCI group: 7.3% vs. 8.6% (*p* = 0.013) No PCI group: 8.2% vs. 8.4% (N.S.)	TIMI major	PCI group: 2.0% vs. 1.1%, *p* < 0.0001 No PCI group: 2.4% vs. 1.0%, *p* < 0.0001
**Long-term Anticoagulant + ASA**				
COMPASS [45]	CV death, MI, stroke	4.1% vs. 4.9% vs. 5.4% Rivaroxaban + ASA vs. ASA alone: *p* < 0.001 Rivaroxaban alone vs. ASA alone: N.S.	Modified ISTH, including fatal bleeding, symptomatic bleeding into a critical organ, bleeding into a surgical site requiring reoperation, and bleeding that led to hospitalization (including presentation to an acute care facility without an overnight stay)	3.1% vs. 2.8% vs. 1.9% Rivaroxaban + ASA vs. ASA alone: *p* < 0.001 Rivaroxaban alone vs. ASA alone: *p* < 0.001

* Main ischemic and bleeding outcomes are listed here separately for easier reading. However, some studies use a combined ischemic and bleeding endpoint for the primary outcome, and/or do not report ischemic and bleeding outcomes separately. Abbreviations: ACS, acute coronary syndrome; ASA, aspirin; BARC, Bleeding Academic Research Consortium; CV, cardiovascular; GUSTO, Global Use of Streptokinase and Tissue plasminogen activator to Open occluded coronary arteries; HR, hazard ratio; ISTH, International Society on Thrombosis and Haemostasis; MI, myocardial infarction; N.S., non-significant; NSTE, non-ST elevation; P2Y_12_i, purinergic P2Y_12_ receptor inhibitor; PCI, percutaneous coronary intervention; PLATO, Study of Platelet Inhibition and Patient Outcomes; RR, relative risk; sCAD, stable coronary artery disease; TIMI, Thrombolysis in Myocardial Infarction.

**Table 4:** Common (a) ischemic and (b) bleeding risk factors for patients with coronary artery disease (CAD) receiving antiplatelet therapy (APT).

**(a) Ischemic Risks**				
**Reference**	**ESC 2020 [80]**			**DAPT [81]**
***Organ System***				
Cardiovascular	Recurrent MI			MI at presentation
	Multivessel disease			Prior PCI or MI
	Multiple stents or treated lesions			Paclitaxel-eluting stent
	Complex revascularization (e.g., left main, bifurcation with ≥2 stents, chronic total occlusion, stented last patent vessel)			Narrow stent (<3 mm diameter)
	History of stent thrombosis			Vein graft stent *
	Early onset or aggressive CAD			Congestive heart failure, * or left ventricular ejection fraction <30% *
	Peripheral arterial disease			
Kidney	Moderate or severe CKD			
***Physical Condition***				
Systemic Condition	Diabetes mellitus			Diabetes mellitus
	Systemic inflammatory diseases (e.g., HIV infection, systemic lupus erythematosus, chronic arthritis)			
Lifestyle				Smoking (within 1 year)
Age				>65 years
				>75 years *
**(b) Bleeding Risks**				
**Reference**	**ARC-HBR ^†^ [60]**		**PRECISE-DAPT [82]**	**CARDIAC [79]**
	**Major**	**Minor**		
***Organ System***				
Blood	Spontaneous bleeding requiring hospitalization or transfusion (past 6 months, or recurrent)	Non-major spontaneous bleeding requiring hospitalization or transfusion (past 12 months)	Previous bleeding	Hemoglobin ↓ from lowest value during hospital stay for PCI
	Hemoglobin <11 g/dL	Hemoglobin 11 − <13 g/dL in men, or 11 − <12 g/dL in women	Hemoglobin <12 g/dL	Hemoglobin <12 g/dL
	Moderate-to-severe thrombocytopenia		White blood cell count ≥ 5 × 10^3^ cells/μL	
	Chronic bleeding diathesis			
Brain	Moderate or severe ischemic stroke (past 6 months)	Non-major ischemic stroke		
	Traumatic (past 12 months) or spontaneous (anytime) intracranial hemorrhage			
Kidney	Severe CKD	Moderate CKD	eGFR ≤ 100 mL/min/1.73 m^2^	eGFR ≤ 60 mL/min/1.73 m^2^
Liver	Liver cirrhosis with portal hypertension			
***Physical Condition***				
Surgery	Major surgery or trauma (< 30 days before PCI)			
	Major surgery while on APT			
Systemic Condition	Malignancy (past 12 months)			
Co-medication	Long-term oral anticoagulant use	Long-term oral NSAID or steroid use		
Age		≥75 years	≥50 years	≥50 years

* Further increased risk. ^†^ A patient is considered to be at HBR when fulfilling ≥1 major or ≥2 minor criteria. Abbreviations: CKD, chronic kidney disease; eGFR, estimated glomerular filtration rate; HIV, human immunodeficiency virus; MI, myocardial infarction; NSAID, non-steroidal anti-inflammatory drug; PCI, percutaneous coronary intervention. “↓”, decrease.

**Table 5:** Key patient considerations for reducing antiplatelet therapy (APT) duration.

Category	Key Consideration	Covered in this Review	
		Section (s)	Illustration
**Ischemic and Bleeding Risk Factors**			
Baseline	Does the patient meet high bleeding risk (HBR) criteria? *	5.1, 5.2	Table 4
Medium-term (1–12 months)	Will the patient’s bleeding risk exceed his/her ischemic risk soon? *	2	Figure 2
**Pharmacological Factors**			
Platelet Reactivity	Is platelet reactivity within normal range? *	5.3 (ABCD-GENE score, genotyping, and/or platelet reactivity test may be useful)	--
Drug–drug Interactions	Is any concurrent medication (present or future) affecting platelet reactivity? ^†^	5.4	--

* “Yes” may favor reducing APT duration. ^†^ May increase or decrease APT effects.

## Data Availability

Not applicable.
